# The *Plasmodium falciparum* eIK1 kinase (PfeIK1) is central for melatonin synchronization in the human malaria parasite. Melatotosil blocks melatonin action on parasite cell cycle

**DOI:** 10.1111/jpi.12685

**Published:** 2020-08-07

**Authors:** Bárbara K.M. Dias, Myna Nakabashi, Marina Rangel Rodrigues Alves, Danielle Pagliaminuto Portella, Benedito Matheus dos Santos, Fahyme Costa da Silva Almeida, Ramira Yuri Ribeiro, Desiree C. Schuck, Alessandro Kappel Jordão, Celia R.S. Garcia

**Affiliations:** ^1^ Departamento de Parasitologia Instituto de Ciências Biomédicas Universidade de São Paulo São Paulo SP Brazil; ^2^ Faculdade de Ciências Farmacêuticas Universidade de São Paulo São Paulo SP Brazil; ^3^ Departamento de Farmácia Faculdade de Farmácia Universidade Federal do Rio Grande do Norte Natal RN Brazil; ^4^ Unidade Universitária de Farmácia Centro Universitário Estadual da Zona Oeste Rio de Janeiro RJ Brazil

**Keywords:** antimalarial, genetic calcium sensor, Melatonin, *Plasmodium falciparum*, synthetic indol

## Abstract

Melatonin and its indoles derivatives are central in the synchronization of malaria parasites. In this research, we discovered that melatonin is unable to increase the parasitemia in the human malaria *Plasmodium falciparum* that lacks the kinase PfeIK1. The PfeIK1 knockout strain is a valuable tool in the screening of indol‐related compound that blocks the melatonin effect in wild‐type (WT) parasite development. The assays were performed by using flow cytometry with simultaneous labeling for mitochondria viability with MitoTracker Deep Red and nucleus staining with SYBR Green. We found that Melatotosil leads to an increase in parasitemia in *P. falciparum* and blocks melatonin effect in the WT parasite. Using microscopy imaging system, we found that Melatotosil at 500 nM is able to induce cytosolic calcium rise in transgenic PfGCaMP3 parasites. On the contrary, the compound Triptiofen blocks *P. falciparum* cell cycle with IC_50_ 9.76 µM ± 0.6, inhibits melatonin action, and does not lead to a cytosolic calcium rise in PfGCaMP3 parasites. We also found that the synthetic indol‐related compounds arrested parasite cycle for PfeIK1 knockout and (WT) *P. falciparum* (3D7) in 72 hours culture assays with the IC_50_ values slighting lower for the WT strain. We concluded that the kinase PfeIK1 is central for melatonin downstream signaling pathways involved in parasite cell cycle progression. More importantly, the indol‐related compounds block its cycle as an upstream essential mechanism for parasite survival. Our data clearly show that this class of compounds emerge as an alternative for the problem of resistance with the classical antimalarials.

## 1 INTRODUCTION

Malaria is the disease caused by parasites from the *Plasmodium* genus and affects millions of people annually. The parasite's life cycle occurs within a mosquito vector, *Anopheles*, and a vertebrate host.^1^

Artemisinin combinatory therapy is used as a first‐line treatment of malaria^2^, ^3^, ^4^ ^.^ Resistance to artemisinin is associated with mutations in the propeller domain of the kelch protein in chromosome 13 of *Plasmodium falciparum*.^3^, ^4^, ^5^, ^6^, ^7^

The increasing parasite resistance to the antimalarials from diverse classes such as chloroquine, piperaquine, mefloquine, artemisinin, and its derivatives^4^, ^7^, ^8^, ^9^, ^10^ creates an urgent need for searching for new ways to combat this lethal infection. Searching for a novel mechanism to break host‐pathogen interaction is a consensus for finding new drugs to combat malaria. Previous work from our group identified compounds from hydroxynaphthoquinones and indole class as a potential antimalarial *in vitro* ^11^, ^12^

Melatonin (N‐acetyl‐ 5‐methoxytryptamine) is the primary hormone secreted by the pineal gland, and its central physiological role is related to hormonal properties^13^, ^14^ ^.^ This hormone is present in a wide variety of organisms, including fungi, macroalgae, protists, plants, and animals^14^, ^15^ ^.^ The secretion of melatonin is related to the light/dark cycle, pointing out that it can stabilize and reinforce circadian rhythm.^13^ Melatonin plays an essential role in the regulation of different physiological processes. It is known to reduce the oxidative stress, regulate gut and retinal physiology,^16^, ^17^, ^18^ inhibit the release of cytochrome b and caspase activation in mitochondria,^19^, ^20^ is associated with immune regulation and offers neuroprotection to neurons.^18^ ^.^ Nevertheless, melatonin is equally related to different pathophysiological processes.^18^, ^21^, ^22^

Melatonin and its derivatives synchronize the intra‐erythrocytic cycle of the human malaria parasite *P. falciparum* as well as the rodent malaria *P. chabaudi*.^23^, ^24^ The downstream signaling in this process involves the activation of a PLC‐IP3 mechanism that leads to cytosolic Ca^2+^ and cAMP increase.^23^, ^25^ The role of indol derivatives compounds to interfere with *P. falciparum* development has been reported and well‐reviewed.^26^.

In this research, we have compared a wild‐type strain (3D7) and a knockout strain of *P. falciparum* eukaryotic initiation factor 2‐alpha kinase 1 (PfeIK1^‐^) and we have searched for the ability of new indol structure‐related compounds to interfere with both parasites cycle. The PfeIK1 kinase (PF3D7_1444500) belongs to the eIF2α kinases family^27^ and was previously described to closely cluster with mammalian GCN2, presenting extensions in both sides of the catalytic domain, anti‐codon domain in the C‐terminal extension and phosphorylates eIF2α in response to amino acid starvation.^28^

eIF2 is the eukaryotic initiation factor 2, composed of three subunits (α, β, and γ). When activated, eIF2 binds to tRNA‐methionine complex forming the ternary complex and initiating protein translation. Activation of eIF2 is GTP dependent, and the activity of a guanine exchange factor is necessary. Phosphorylation of eIF2α results in repression of general protein translation and is a well‐known mechanism of stress response, since mRNA encoding for proteins from stress response still translated.^29^, ^30^, ^31^ Our studies lead us to conclude that synchronization upon melatonin requires not only the PfPK7^32^ but also involves PfeIK1. Moreover, indol‐related compounds are equally involved in the cell cycle progression, which explains synthetic indol‐related compounds' ability to block parasite proliferation in PfeIK1 knockout parasite strain.

## 2 EXPERIMENTAL SECTION

### 2.1 Chemistry

The chemical reagents and all solvents used in this study were purchased from Merck Millipore ‐Brazil. Melatonin was purchased from Sigma Aldrich Brazil LTDA. Melting points were determined with a Fisher Johns instrument and are uncorrected. Infrared (IR) spectra were recorded on an ABB FTLA2000‐100 spectrophotometer in KBr pellets (Quebec, Canada). NMR spectra, unless otherwise stated, were obtained in deuterated Me_2_SO‐d_6_ using a Varian Unity Plus 300 MHz spectrometer. Chemical shifts (δ) are expressed in ppm and the coupling constant (*J*) in Hertz. Reactions were routinely monitored by thin‐layer chromatography (TLC) on silica gel pre‐coated F254 Merck plates. Column chromatography was performed on silica gel flash from across. The developed chromatograms were viewed under ultraviolet light at 254 nm. The compound **2** has been synthesized using Venkatachalam and co‐workers protocol.^33^

#### 2.1.1 *N*‐(2‐(1*H*‐indol‐3‐yl) ethyl)benzenesulfonamide (Tripbenz (**4**))

Obtained in 89% yield as a pale yellow solid; m.p. 103‐106°C; IR (KBr) υ_max_ (cm^−1^) 3434, 3320 (N‐H); 1153 (S = O); ^1^H‐NMR (300 MHz, DMSO‐*d*_6_) δ 2.77‐2.80 (m, 2H, CH_2_); 2.98‐3.03 (m, 2H, CH_2_); 6.92‐6.96 (m, 1H); 7.02‐7.06 (m, 1H); 7.10‐7.11 (m, 1H); 7.30‐7.38 (m, 2H); 7.54‐7.64 (m, 3H); 7.74‐7.77 (m, 1H); 7.79‐7.82 (m, 2H); 10.8 (bs, 1H, N‐H) ppm. ^13^C‐NMR (75 MHz, DMSO‐d_6_) δ_c_ 140.6; 136.2; 132.3; 129.2; 127.0; 126.5; 123.0; 121.0; 118.3; 118.0; 111.4; 111.0; 43.5; 25.4 ppm.

#### 2.1.2 *N*‐(2‐(1*H*‐indol‐3‐yl)ethyl)‐4‐methylbenzenesulfonamide (Triptosil (**5**))

Obtained in 67% yield as a pale yellow solid; m.p. 105‐109°C; IR (KBr) υ_max_ (cm^−1^) 3406, 3280 (N‐H); 1153 (S = O); ^1^H‐NMR (300 MHz, DMSO‐*d*_6_ δ 2.36 (s, 3H, CH_3_); 2.75‐2.79 (m, 2H, CH_2_); 2.94‐2.99 (m, 2H, CH_2_); 6.92‐6.96 (m, 1H); 7.01‐7.05 (m, 1H); 7.09‐7.10 (m, 1H); 7.29‐7.37 (m, 4H); 7.63‐7.68 (m, 3H); 10.8 (bs, 1H, N‐H) ppm.^13^C‐NMR (75 MHz, DMSO‐*d*_6_) δ_c_ 142.5; 137.7; 136.2; 129.6; 126.7; 126.5; 122.9; 120.9; 118.3; 117.9; 111.4; 111.0; 43.5; 25.4; 21.0 ppm.

#### 2.1.3 *N*‐(2‐(1*H*‐indol‐3‐yl) ethyl)‐5‐bromothiophene‐2‐carboxamide (Triptiofen (**6**))

Obtained in 64% yield as a pale yellow solid; m.p. 113‐117°C; IR (KBr) υ_max_ (cm^−1^) 3402, 3273 (N‐H); 1620 (C = O); ^1^H‐NMR (300 MHz, DMSO‐*d*_6_) *δ* 2.91‐2.95 (m, 2H, CH_2_); 3.47‐3.53 (m, 2H, CH_2_); 6.95‐6.99 (m, 1H); 7.04‐7.08 (m, 1H); 7.09‐7.10 (m, 1H); 7.12‐7.14 (m, 1H); 7.16‐7.17 (m, 1H); 7.32‐7.34 (m, 1H); 7.56‐7.57 (m, 1H); 7.72‐7.73 (m, 1H); 8.64 (t, 1H, N‐H, *J *= 5.6 Hz); 10.8 (bs, 1H, N‐H) ppm. ^13^C‐NMR (75 MHz, DMSO‐*d_6_*) δ_c_ 161.0; 140.3; 136.2; 130.5; 127.8; 127.7; 127.2; 122.6; 121.0; 118.3; 118.2; 111.7; 111.4; 40.1; 25.3 ppm.

#### 2.1.4 *N*‐(2‐(1*H*‐indol‐3‐yl)ethyl)furan‐2‐carboxamide (Tripfuroila (**7**))

Obtained in 31% yield as a pale yellow solid; m.p. 145‐148°C; IR (KBr) υ_max_ (cm^−1^) 3263 (N‐H); 1614 (C = O); ^1^H‐NMR (300 MHz, DMSO‐*d*_6_) δ 2.89‐2.92 (m, 2H, CH_2_); 3.46‐3.51 (m, 2H, CH_2_); 6.60‐6.61 (m, 1H); 6.95‐6.98 (m, 1H); 7.03‐7.05 (m, 1H); 7.06‐7.07 (m, 1H); 7.15‐7.16 (m, 1H); 7.31‐7.33 (m, 1H); 7.55‐7.57 (m, 1H); 7.80‐7.81 (m, 1H); 8.47 (t, 1H, N‐H, *J* = 5.6 Hz); 10.8 (bs, 1H, N‐H) ppm.^13^C‐NMR (75 MHz, DMSO‐*d_6_*) δ_c_ 157.7; 148.1; 144.8; 136.2; 127.2; 122.6; 120.9; 118.3; 118.2; 113.1; 111.8; 111.7; 111.4; 39.3; 25.3 ppm.

#### 2.1.5 *N*‐(2‐(1*H*‐indol‐3‐yl)ethyl)cyclohexanecarboxamide (Tripcicloexila (**8**))

Obtained in 64% yield as a pale yellow solid; m.p. 77‐83°C; IR (KBr) υ_max_ (cm^−1^) 3342; 3273 (N‐H); 1626 (C=O); ^1^H‐NMR (300 MHz, DMSO‐*d*_6_) δ 1.13‐1.37 (m, 5H); 1.62‐1.66 (m, 5H); 2.01‐2.09 (m, 1H); 2.76‐2.80 (m, 2H); 3.25‐3.33 (m, 4H); 6.93‐6.98 (m, 1H); 7.02‐7.07 (m, 1H); 7.10‐7.11 (m, 1H); 7.30‐7.33 (m, 1H); 7.50‐7.53 (m, 1H); 7.74‐7.78 (m, 1H); 10.8 ppm. (bs, 1H, N‐H) ^13^C‐NMR (75 MHz, DMSO‐*d*_6_) δ_c_ 175.0; 136.2; 127.2; 122.5; 120.8; 118.1; 111.9; 111.3; 44.0; 29.2; 25.5; 25.3; 25.2 ppm.

#### 2.1.6 *N*‐(2‐(5‐methoxy‐1*H*‐indol‐3‐yl)ethyl)benzenesulfonamide (Melatobenz (**9**))

Obtained in 61% yield as a pale yellow solid; m.p. 136‐140°C; IR (KBr) υ_max_ (cm^−1^) 3296 (N‐H); 1172 (S=O); ^1^H‐NMR (300 MHz, DMSO‐*d*_6_) δ 2.89‐2.93 (m, 2H, CH_2_); 3.49‐3.56 (m, 2H, CH_2_); 3.71 (s, 3H, CH_3_); 6.68‐6.72 (m, 1H); 7.04‐7.05 (m, 1H); 7.12‐7.13 (m, 1H); 7.20‐7.23 (m, 1H); 7.41‐7.53 (m, 4H); 7.82‐7.85 (m, 2H); 8.58 (t, 1H, N‐H, *J* = 5.6 Hz); 10.6 (bs, 1H, N‐H) ppm. ^13^C‐NMR (75 MHz, DMSO‐*d*_6_) δ_c_ 166.1; 153.0; 134.7; 131.3; 131.0; 128.2; 127.6; 127.1; 123.3; 112.0; 111.8; 111.0; 100.1; 55.2; 40.2; 25.2 ppm.

#### 2.1.7 *N*‐(2‐(5‐methoxy‐1*H*‐indol‐3‐yl)ethyl)‐4‐methylbenzenesulfonamide (Melatotosil (**10**))

Obtained in 57% yield as a pale yellow solid; m.p. 140‐143°C; IR (KBr) υ_max_ (cm^−1^) 3392 (N‐H); 1151 (S = O); ^1^H‐NMR (300 MHz, DMSO‐*d*_6_ δ 2.36 (s, 3H, CH_3_); 2.71‐2.76 (m, 2H, CH_2_); 2.91‐2.98 (m, 2H, CH_2_); 3.72 (s, 3H, CH_3_); 6.66‐6.70 (m, 1H); 6.83‐6.84 (m, 1H); 7.04‐7.05 (m, 1H); 7.18‐7.20 (m, 1H); 7.34‐7.37 (m, 2H); 7.61‐7.68 (m, 3H); 10.6 (bs, 1H, N‐H) ppm. ^13^C‐NMR (75 MHz, DMSO‐*d_6_*) δ_c_ 152.9; 142.5; 137.7; 131.3; 129.6; 127.3; 126.5; 123.5; 112.0; 111.0; 110.7; 99.7; 55.3; 43.3; 25.4; 20.9 ppm.

#### 2.1.8 *N*‐(2‐(5‐methoxy‐1*H*‐indol‐3‐yl)ethyl)furan‐2‐carboxamide (Melatofuroila (**11**))

Obtained in 40% yield as a pale yellow solid; m.p. 66‐70°C; IR (KBr) υ_max_ (cm^−1^) 3361; 3255 (N‐H); 1624 (C = O); ^1^H‐NMR (300 MHz, DMSO‐*d*_6_) δ 2.85‐2.90 (m, 2H, CH_2_); 3.44‐3.51 (m, 2H, CH_2_); 3.72 (s, 3H, CH_3_); 6.59‐6.61 (m, 1H); 6.68‐6.71 (m, 1H); 7.04‐7.07 (m, 2H); 7.11‐7.12 (m, 1H); 7.19‐7.22 (m, 1H); 7.79‐7.80 (m, 1H); 8.44 (t, 1H, N‐H, *J* = 5.8 Hz); 10.8 (bs, 1H, N‐H) ppm. ^13^C‐NMR (75 MHz, DMSO‐*d*_6_) δ_c_ 157.7; 153.0; 148.1; 144.8; 131.4; 127.6; 123.3; 113.1; 112.0; 111.8; 111.6; 111.1; 100.1; 55.3; 39,4; 25.3 ppm.

#### 2.1.9 *N*‐(2‐(5‐methoxy‐1*H*‐indol‐3‐yl)ethyl)cyclohexanecarboxamide (Melatocicloexil (**12**))

Obtained in 60% yield as a pale yellow oil; IR (KBr) υ_max_ (cm^−1^) 3408; 3280 (N‐H); 1638 (C = O); δ 1.13‐1.37 (m, 5H); 1.59‐1.69 (m, 5H); 2.02‐2.09 (m, 1H); 2.72‐2.77 (m, 2H); 3.25‐3.29 (m, 2H); 3.75 (s, 3H, CH_3_); 6.68‐6.72 (m, 1H); 6.99‐7.00 (m, 1H); 7.06‐7.07 (m, 1H); 7.19‐7.22 (m, 1H); 7.70‐7.74 (m, 1H); 10.6 ppm. ^13^C‐NMR (75 MHz, DMSO‐*d*_6_) δ_c_ 175.0; 152.9; 131.3; 127.5; 123.2; 111.9; 111.7; 111.0; 100.2; 55.3; 44.0; 39.2; 29.2; 25.5; 25.3 ppm.

### 2.2 Synthesis of Indole Compounds

In a recent study published by Schuck et al,^12^ compound (**1**) was identified as a prototype with potential antimalarial activity against *P. falciparum* with IC_50_ 2.93 µM. This compound, unlike the others prepared in that paper, has an aromatic ring as a substituent on the amide moiety. This result motivated our research group to continue the investigation of indole derivatives through the structural modification of compound (**1**), with the substitution of the benzene aromatic ring with heterocyclic and cyclohexane rings. In addition, the exchange of carbonyl by the sulfonyl group was studied to verify the optimization of antimalarial activity and the influence of the substitution pattern in these new compounds Tripbenz (**4**), Triptosil (**5**), Triptiofen (**6**), Tripfuroila (**7**), Tripcicloexil (**8**), Melatobenz (**9**), Melatotosil (**10**), Melatofuroila (**11**), and Melatocicloexil (**12**).

Methoxytryptamine^33^ or tryptamine an aqueous solution of NaOH at 0°C and chloroform were reacted with the appropriate acyl chloride, benzene sulfonyl chloride, or toluene sulfonyl chloride to give the desired indoles. The general procedure for the preparation of the indole derivatives (**4)**‐(**12)** was described by Schuck et al^12^

### 2.3 *Plasmodium falciparum*: *in vitro* culture

*Plasmodium falciparum* 3D7 and PfeIK1^‐^ parasites were maintained in RPMI media supplemented with A^+^ human erythrocytes (parasitemia: up to 5%, hematocrit: 2%) cultivated in RPMI‐1640 media (GIBCO) supplemented with 0.04% gentamicin sulfate, 0.05 % hypoxanthine, and 0.5% Albumax II (Gibco). PfeIK1^‐^ was cultivated with 2.5 µg/mL of blasticidin for selection. PfeIK1^‐^ parasites^28^ were kindly donated by Christian Doerig from Monash University.

### 2.4 Quantitative real‐time PCR

For these experiments, *P. falciparum* 3D7 culture was synchronized with 5% sorbitol and parasites collected in the three stages: ring (14 hours); trophozoite (30 hours); and schizont (44 hours), at 4% parasitemia. Parasites were treated with 100 nM Melatonin or 0.00005% ethanol (control) and left for 4 hours before RNA extraction with Trizol (Invitrogen). RNA purified with ionic exchange columns with DNAse using RNAse‐free DNAse set (Qiagen) and RNeasy Mini Kit (Qiagen) and quantified in a UV‐Vis NanoDrop 2000c (Thermo Scientific). Three independent experiments were performed in triplicates.

cDNA syntheses were performed using random primers and Superscript II reverse transcriptase (Invitrogen) according to the manufacturer’s protocol. SYBR Green (Applied Biosystems) was used in quantitative real‐time PCR on a 7300 Real‐Time PCR System (Applied Biosystems). Amplification was carried out as follow: on step 50°C for 2 min and 95°C for 10 min for enzyme activation followed by 40 cycles of 95°C for 0.15 min for denaturation and 60°C for 1 min for annealing/extension. The primers used were as follows: PF14_0423‐Fwd ACGCACTCAAACCAATCAACTTT and PF14_0423‐Rvs GTTTATTAACTCCGCTTGGTCCAT.

Changes in relative expression were determined by 2^‐ΔΔCt^ or transforming the threshold cycle in absolute number of mRNA. We performed statistical analysis with ΔΔCt values in log2 with relative expression of the genes normalized to housekeeping gene seryl‐tRNA synthetase.

### 2.5 Effect of indole compounds in parasitemia

Red blood cells infected (iRBC) with 3D7 or PfeIK1^‐^ (iRBC) with 1% initial parasitemia and 2% hematocrit were incubated with 500 nM of the synthetic indole compounds or 100 nM Melatonin for 48 hours at 37°C under mixture of gases.^12^ The control group received 0.0013% of solvent dimethyl sulfoxide (DMSO).

To evaluate the effect of the compounds on melatonin action on parasitemia, iRBC were incubated with 500 nM of compounds for 10 minutes and then along with 100 nM of melatonin for 48 hours at 37°C under mixture of gases.

After 48 hours, iRBC were double‐stained^34^ with nucleic acid marker SYBR Green I (1X) and mitochondria marker, based on membrane potential, MitoTracker Deep Red (50 nM), and incubated for 20 minutes at 37°C.

Final parasitemia was obtained by flow cytometer using an Accuri C6 flow cytometer (Becton Dickinson), collecting 10^4^ cells. Parasitemia was determined by dot plots using FlowJo 5 software.

### 2.6 *In vitro* growth assay and flow cytometry analysis

Red blood cells infected with *P. falciparum* 3D7 or PfeIK1^‐^ (iRBC) with 0.3 % initial parasitemia and 1% hematocrit were incubated with different concentrations of compounds Tripbenz (**4**), Triptosil (**5**), Triptiofen (**6**), Tripfuroila (**7**), and Tripcicloexil (**8**) ranging from 0.107 to 110 µM, and compounds Melatobenz (**9**), Melatotosil (**10**), Melatofuroila (**11**), and Melatocicloexil (**12**) ranging from 0.1 to 100 µM for 72 hours at 37°C under mixture of gases. The control group was treated with solvent dimethyl sulfoxide (DMSO) (v/v), with the highest concentration of solvent 0.14%. Chloroquine was used as positive control with concentrations ranging from 0.244 to 250 nM. Parasitemia was determined from dot plots (side scatter versus fluorescence) of at least 10^3^ cells acquired on a FACSCalibur flow cytometer using CELLQUEST software (Becton Dickinson) or an Accuri C6 flow cytometer (Becton Dickinson). Initial gating was carried out with unstained, uninfected erythrocytes to account for erythrocyte auto fluorescence. The concentration responsible for 50% inhibition (IC_50_) was determined from the compound concentration‐response curve determined with the software GraphPad Prism 6.

For assays with antimalarials, iRBC were incubated with different concentrations of Atovaquone ranging from 0.02 to 24 nM, or Artemisinin 0.15‐160 nM.

After 72 hours, iRBC were stained with nucleic acid marker SYBR Green I (1X) (Invitrogen) and mitochondria marker based on membrane potential MitoTracker Deep Red (50 nM) (Invitrogen), and incubated for 20 minutes at 37°C. Final parasitemia was obtained by flow cytometer using a BD Accuri C6 Flow Cytometer, collecting 10^4^ cells. Parasitemia was determined by dot plots using FlowJo 5 software.

At least three independent experiments were performed in triplicate to calculate the IC_50_ values for each drug.

### 2.7 Microscopy evaluation of blood‐stage development of *P. falciparum* (3D7)

*Plasmodium falciparum* (3D7) cultures were synchronized by three rounds of 5% sorbitol treatment for 1 hour.^35^ Parasites at ring stage with initial 1% parasitemia and 2% hematocrit were incubated with Triptiofen (**6**) at concentrations of 1 and 10 µM in 24‐well plates for 48 hours at 37°C under mixture of gases. The DMSO solvent (0.1%) was used as a control. Smears of infected erythrocytes stained with Giemsa were performed at regular intervals (0, 12, 24, 36 and 48 hours after treatment).^36^ Parasite images were acquired under a light microscope (Zeiss) with 100× magnification to assess the parasite's blood development stages. The parasitemia at the end of the experiment was quantified for comparison between groups. Three independent experiments were performed in triplicate. Data were analyzed using software GraphPad Prism 6.

### 2.8 Cytotoxicity Assay in HEK293 cells

To evaluate toxicity of the new indole compounds in mammalian cells, Human Embryonic Kidney cells (HEK293) were maintained in 75 cm^2^ culture flasks (Greiner Bio‐One) at 37°C, 5%CO_2_ in Dulbecco’s Minimum Essential Media (DMEM), supplemented with 10% fetal bovine serum, NaHCO33.7 g/L (Sigma), 100 U/mL penicillin/, and 100 μg/mL streptomycin in monolayers.

Cells were plated in flat‐bottom 96‐well plates in a density of 10^4^ cells per well and incubated for 24 hours in 150 µL media at 37°C, 5%CO_2._ Cells were then treated with different concentrations of each compound, ranging from 0.107 to 110 µM in 200 µL DMEM for 72 hours at 37°C and 5%CO_2._ Cells treated with DMSO (v/v) with the highest concentration of solvent 0.14% were used as control.

After incubation, 40 µL of MTT reagent (solution 5 mg/mL in PBS) was added to each well, and cells were incubated for further 3 hours at 37°C and 5% CO_2._ The media were removed and 100 µL of DMSO was added in each well. Plates were agitated in a shaker for 10 minutes to dissolve precipitates. Absorbance was read at 570 nM in a FlexStation 3 (Molecular Devices). Six experiments were performed independently in triplicates.

### 2.9 Calcium assay

Calcium assays were performed using the transgenic *P. falciparum* GCaMP3 parasites.^37^ PfGCaMP3 cultures were synchronized at least three times with 5% sorbitol before starting the experiments, and parasitemia was maintained between 5% and 8%. PfGCaMP3 parasites were cultivated with 10 nM/mL of WR for selection. Parasites were isolated with saponin (0.016%) and collected by centrifugation 8000 rpm, subsequently washed twice with buffer M, and allowed to settle in a four‐well plate for microscope coated with poly‐l‐lysine for 30 minutes at 37°C with 5% CO_2_ in buffer M (16 mM NaCl, 5.4 mM KCl, 0.8 mM MgSO4, 5.5 mMD‐glucose, 50 mM MOPS, and 2 mM CaCl2, pH 7.2). The compounds used for measuring calcium assays were as follows: Melatotosil and Triptiofen solubilized in DMSO.

Images were captured using a Nikon Eclipse Ti2 inverted microscope with FITC filter. Images were collected every 10 seconds for 5 minutes, with NIS‐Elements Ar 5.11.01 from Nikon using 60× lens. All experiments were performed at least three independent times in duplicates.

## 3 RESULTS

### 3.1 Effect of melatonin in the expression of *pfeik1* in 3D7 parasites

To better understand the role of regulation of protein translation in the melatonin effect in the parasite erythrocytic cycle, we investigated whether melatonin treatment could modulate gene expression of *pfeik1*. For this purpose, synchronous 3D7 parasites in ring, trophozoite, and schizont stages were treated with 100 nM melatonin for 4 hours.

**Figure 1:**
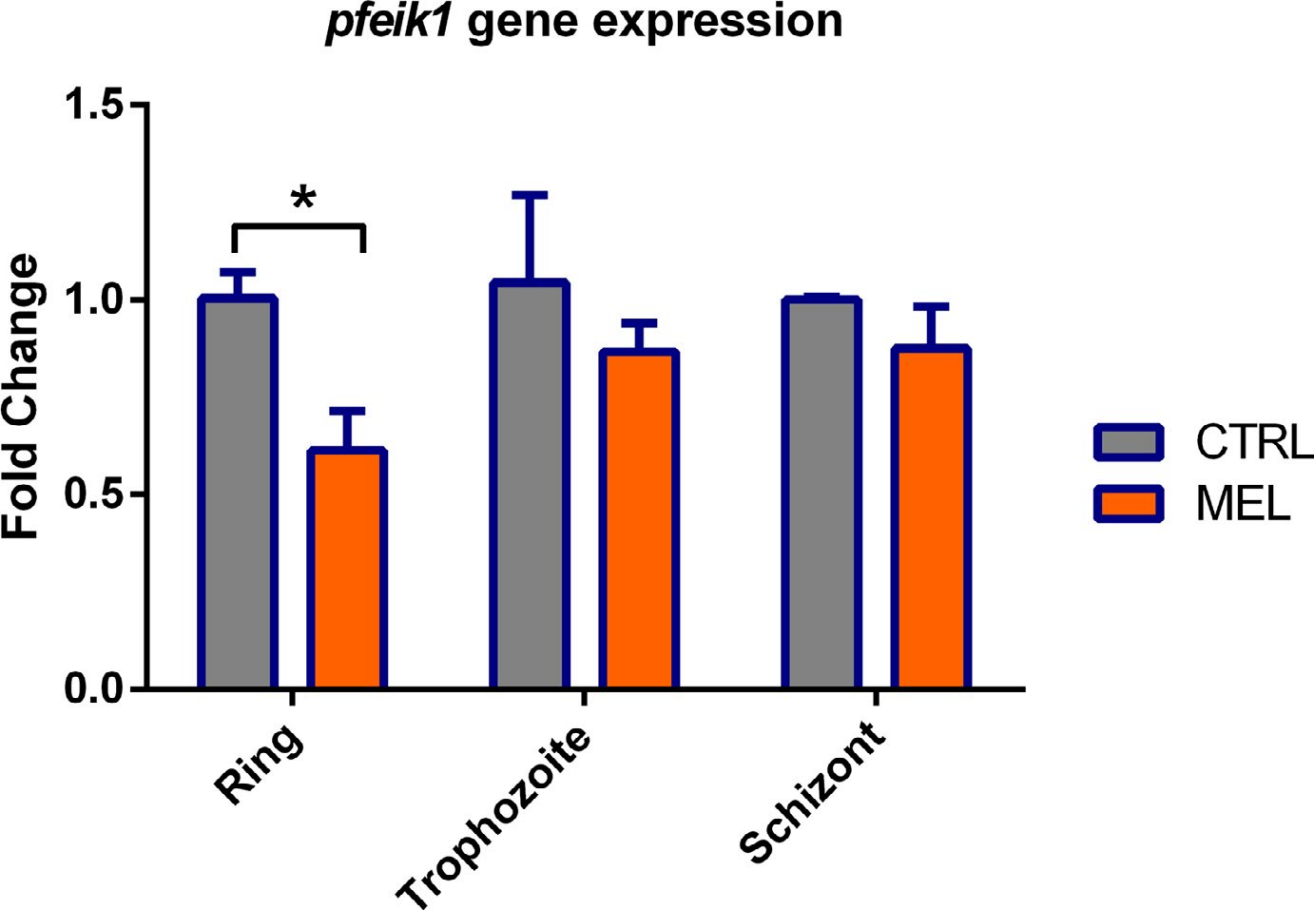
Effect of melatonin in *pfeik1* gene expression during *Plasmodium falciparum* 3D7 erythrocytic cycle. Erythrocytes infected with *P. falciparum* parasites in the stages of ring (14 h), trophozoite (30 h), and schizont (40 h) were treated with 100 nM melatonin for 4 h, and *pfeik1* expression was analyzed and compared to parasites that received only the solvent. The gene expression was normalized to the housekeeping gene seryl‐tRNA synthetase. Experiments were performed in three independent biological replicates. Statistical analysis was performed with ΔΔCt values in log2. “*” indicates a significant difference. Student’s t test followed by Dunnett’s test (*P ≤ .05; **P ≤ .01; ***P ≤ .001; ****P ≤ .0001)

Figure 1 shows that ring stage parasites showed a 40% decrease in *pfeik1* expression with melatonin treatment compared to control. However, at the trophozoite and schizont stages, no significant difference was observed in parasites treated with melatonin or solvent.

Phosphorylation of eIF2 occurs mainly in response to cell stress and results in an arrest in protein translation,^30^ which could result in an arrest of cell cycle progression.

The melatonin effect in *pfeik1* expression in ring stage parasites corroborates with the evidence that melatonin would accelerate the erythrocytic parasite cycle, leading to an increase of parasites in the mature form schizont and consequently, to a rise in parasitemia previously described in the literature^24^, ^38^

### 3.2 Effect of indole compounds in *P. falciparum* 3D7 and PfeIK1 knockout parasites

**Figure 2:**
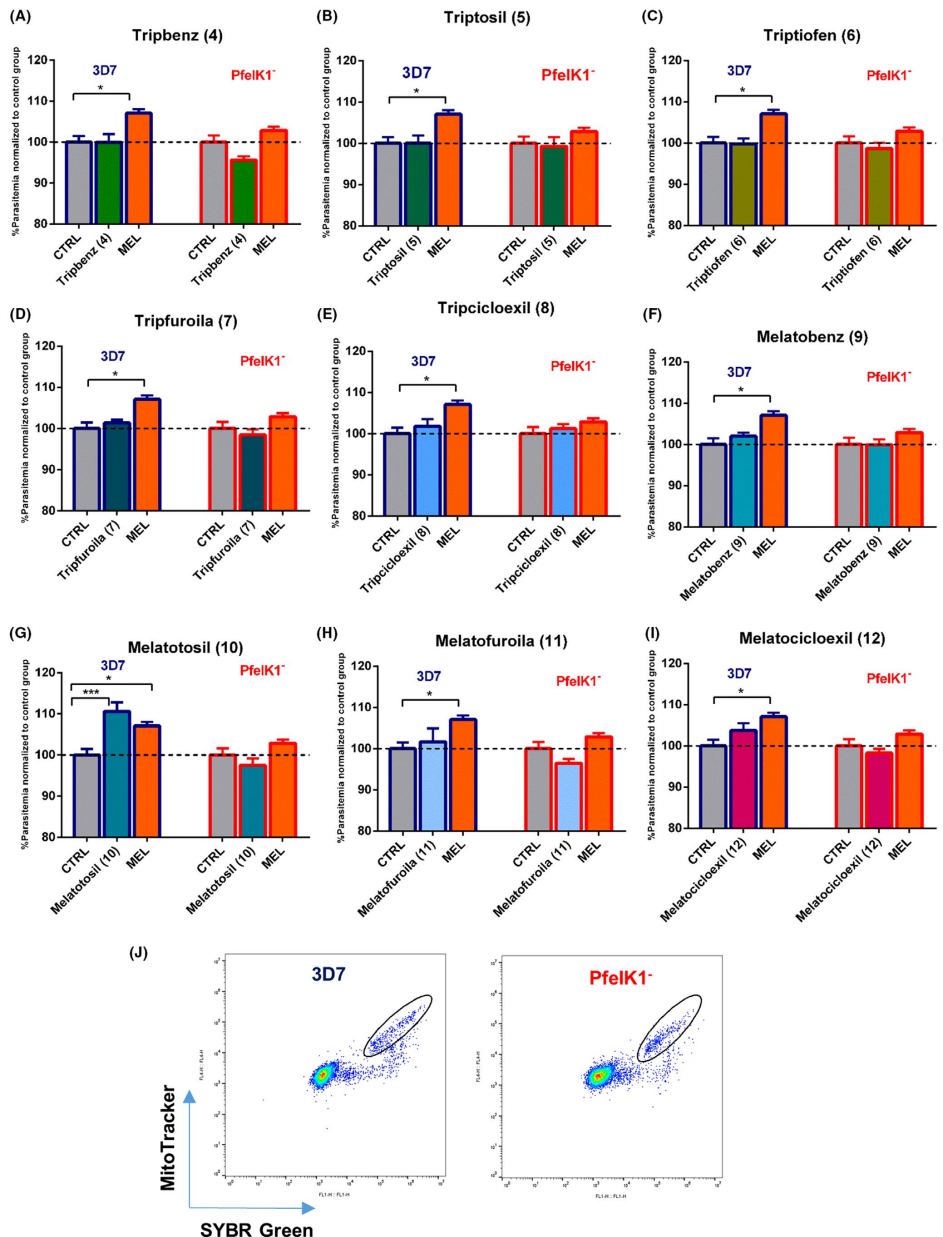
Effect of indole compounds on *Plasmodium falciparum* parasitemia. Erythrocytes infected with *P. falciparum* 3D7 (Blue) or PfeIK1^‐^ (Red) were incubated for 48 h with 500 nM of each indole compoundsTripbenz (**4**) (A), Triptosil (**5**) (B), Triptiofen (**6**) (C), Tripfuroila (**7**) (D), Tripcicloexil (**8**) (E), Melatobenz (**9**) (F), Melatotosil (**10**) (G), Melatofuroila (**11**) (H), and Melatocicloexil (**12**) (I) or with 100 nM melatonin (MEL). Parasites were stained with MitoTracker Deep Red and SYBR Green I and final parasitemia was determined by flow cytometer using a BD Accuri C6 (BD Bioscience), representative dot plots are shown in J. Data represent the percentage of parasitemia normalized to the control group treated with solvent (DMSO). Experiments were performed three independent times in triplicates. Error bars represent the SEM. “*” Indicates significant difference. One‐way ANOVA followed by Dunnett’s test (*P ≤ .05; **P ≤ .01; ***P ≤ .001; ****P ≤ .0001)

To investigate the potential role of the *P. falciparum* kinase PfeIK1 in the melatonin signal transduction pathways of parasite synchronization, we have tested the ability of melatonin to increase parasitemia in parasite strain lacking PfeIK1 expression (PfeIK1^‐^). To evaluate the parasitemia of PfeIK1^‐^ parasites submitted to melatonin incubation, a culture of infected erythrocytes was incubated with 100 nM of the hormone for 48 hours. To evaluate the final parasitemia of PfeIK1 parasites after the incubation with melatonin, we have used flow cytometry and a double‐label procedure for staining DNA with SYBR green staining^34^ and mitochondria membrane potential with MitoTracker Deep Red to ensure to analyze only viable parasites. Figure 2J shows typical dot plots for the analysis of parasitemia while Figure 2 shows that treatment with 100 nM melatonin for 48 hours increased parasitemia in approximately 10% in wild‐type parasites strain of *P. falciparum (*3D7) but showed no effect in parasitemia in the knockout strain, PfeIK1^‐^ . We further investigated the ability of a series of new indol‐synthetic compounds to interfere with parasitemia of both wild‐type and PfeIK1^‐^ *P. falciparum* parasites as the new finding represents an interesting tool to dissect indol‐signaling pathways and to interfere with parasite cell cycle progression.

We investigated the ability of synthetic compounds to interfere with parasitemia by incubating the synthetic compounds at 500 nM concentration for 48 hours with the wild‐type parasites culture of *P. falciparum* (3D7). Figure 2 shows that the final parasitemia after the incubation of parasites (3D7) with the following compounds: Tripbenz (**4**) 99.96% (±2.03), Triptiofen (**6**), 99.81% (±1.30), Tripfuroila (**7),** 101.4% (±0.76), Tripcicloexil (**8**), 101.8% (±1.74), Melatobenz (**9**), 102.0% (±0.84), Melatofuroila (**11**) 101.7% (±3.55), and Melatocicloexil (**12**) 103.7% (±1.81) has no significant effect on parasitemia when compared to control group.

Melatotosil (**10**), unlikely the previous compounds tested, was able to increase parasitemia in *P. falciparum* 3D7 parasites by 10% (±2.19) when compared to the control group, treated with the solvent, (Figure 2G). Strikingly, this effect was not observed in experiments with *P. falciparum* parasites lacking PfeIK1, as the final parasitemia was found to be 97.5% (±1.67) after treating these parasites with Melatotosil (**10**) (Figure 2G). We further investigated the relevance of methoxy group in C5 in the structure of Melatotosil in the ability of the compound to modulate the malaria parasite cycle. We therefore removed methoxy group in C5 in the synthetic compound Triptosil (**5**). Our data show clearly the relevance of removing this group in C5 of the indole ring, rendering Triptosil unable to affect the parasitemia in *P. falciparum* 3D7 parasites (100% ± 1.91) (Figure 2B). As expected the knockout strain parasites, PfeIK1^‐^ does not show any change in parasitemia after incubating these parasites with Triptosil (99.29 ± 2.18).

Moreover, following the data obtained for parasites lacking PfeIK1, treatment with melatonin showed no significant effect on parasitemia (102.3% ± 0.88), similar results were obtained by incubating these parasites with the new indole compounds. Parasitemia (normalized to control) obtained for each compound was as follow: Tripbenz (**4**) 95.64% (±0.89), Triptiofen (**6**) 98.65% (±1.40), Tripfuroila (**7**) 98.48% (±1.29), Tripcicloexil (**8**)101.2% (±1.08), Melatobenz (**9**) 99.95% (±1.26), Melatofuroila (**11**) 97.08% (±0.98), and Melatocicloexil (**12**) 98.29% (±1.00) (Figure 2).

These results clearly show that the kinase PfeIK1 is a molecular component of the downstream melatonin‐signaling pathways, and the lack of this kinase abolishes the increase in parasitemia triggered by melatonin.

### 3.3 Antimalarial activity of indole compounds and IC_50_ values determination.

**Figure 3:**
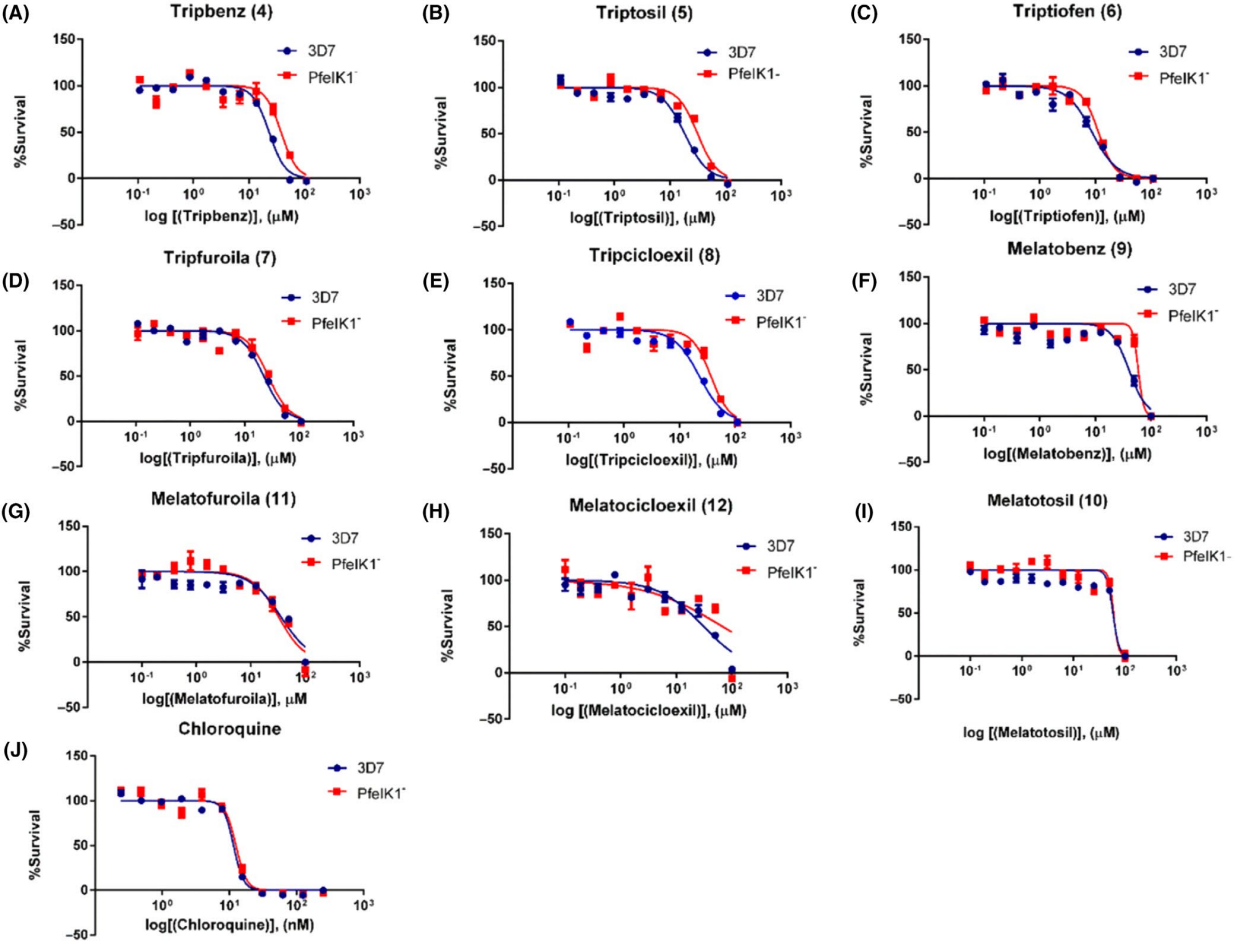
Determination of antiplasmodial activity of synthetic indole compounds in 3D7 and PfeIK1^‐^. Growth survival curves of blood stage *Pasmodium falciparum* with concentrations ranging from 0.107 to 110 µM for compounds Tripbenz (**4**), Triptosil (**5**), Triptiofen (**6**), Tripfuroila (**7**), and Tripcicloexil (**8**), for 3D7 (Blue) or PfeIK1^‐^ (Red), or 0.100 to 100 µM for compounds Melatobenz (**9**), Melatofuroila (**11**), and Melatocicloexil (**12**). Chloroquine was used as positive control with concentrations ranging from 0.244 to 250 nM. Experiments were performed three independent times in triplicates. Error bars represent the SEM

In the next step, we search for the ability of synthetic indole compounds to interfere with the parasite growth in (*P. falciparum* 3D7 and PfeIK1^‐^) *in vitro* assays by incubating them for 72 hours in a range of different concentrations and evaluating the final parasitemia using the same approach as above, the flow cytometry after double‐labeling. We were able to construct survival curves (Figure 3) and to determine the IC_50_ for each compound.

We first tested five compounds (Tripbenz (**4**), Triptosil (**5**), Triptiofen (**6**), Tripfuroila (**7**), and Tripcicloexil (**8**)) containing a bulky chain in C3 of the indole ring. The compounds tested showed an antimalarial activity with the IC_50_ values in the micromolar range (Table 1).

**Table 1:** Indole derivatives: structure and *in vitro* antiplasmodial activity

Compound	Structure	IC_50_ (µM) 3D7 strain	IC_50_ (µM) PfelK1^‐^ strain
Tripbenz (**4**)	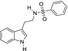	18.65 ± 2.50	36.84 ± 3.44
Triptosil (**5**)	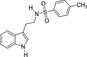	19.79 ± 5.33	30.60 ± 1.55
Triptiofen (**6**)	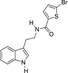	9.76 ± 0.60	11.80 ± 0.60
Tripfuroila (**7**)	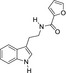	17.12 ± 2.60	23.64 ± 2.20
Tripcicloexil (**8**)	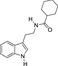	28.03 ± 4.07	37.61 ± 2.72
Melatobenz (**9**)	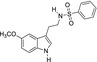	38.52 ± 1.57	57. 76 ± 1.31
Melatotosil (**10**)	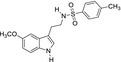	No activity	No activity
Melatofuroila (**11**)	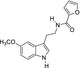	30.95 ± 4.92	31.95 ± 0.46
Melatocicloexil (**12**)	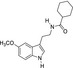	35.22 ± 4.14	47.55 ± 6.43
Atovaquone		0.40 nM ± 0.08	0.47 nM ± 0.07
Artemisinin		13.69nM ± 2.1	18.84 nM ± 3.6
Chloroquine		9.90 nM ± 1.00	11.33 ± 0. 65

Tripbenz (**4**) showed an IC_50_ of 18.65 µM for 3D7 parasites and 36.84 µM PfeIK1^‐^ parasites (Table 1). Triptosil (**5**) showed an IC_50_ 19.79 µM for 3D7 and 30.60 µM for PfeIK1^‐^ . The results with Tripbenz (**4**) and Triptosil (**5**) suggest that the substituent benzene aromatic ring on the carbonic side chain is important for antimalarial activity. When the aromatic ring is replaced by the cyclohexyl ring in Tripcicloexil (**8**), the IC_50_ values increase (28.03 µM in 3D7 and 37. 61 µM in PfeIK1^‐^) (Table 1) corroborating with this conclusion.

With the benzene aromatic ring, the classic bioisosteric aromatic benzene ring exchange was performed in Tripbenz (**4**) and Triptosil (**5**) by thiophene and furan rings, preparing Triptiofen (**6**) and Tripfuroila (**7**), respectively, which presented even better results. Triptiofen (**6**) exhibited IC_50_ value of 9.76 µM in wild‐type parasites 3D7 and 11.80 µM in parasites lacking PfeIK1^‐^ (Table 1), followed by Tripfuroila (**7**) that presented IC_50_ 17.12 µM for 3D7 and 23.64 µM for PfeIK1^‐^ (Table 1).

Furthermore, we tested new compounds adding a methoxy group in C5 of the indole ring and presenting the same substitutions in C3. When comparing the results of Melatobenz (**9**) (IC_50_ 38.52 µM in 3D7 and 57.76 µM in PfeIK1^‐^), Melatofuroila (**11**) (30.95 µM in 3D7 and 31.95 µM in PfeIK1^‐^) and Melatocicloexil (**12**) (35.22 µM in 3D7 and 47.55 µM in PfeIK1‐) with the methoxy substituent on the C5 of the indole ring, the activity of these compounds is lower (Table 1).

As expected for this series of new compounds, the methoxy group at position C5 of the indolic ring resulted in the Melatotosil (**10**) without antimalarial activity (Table 1). Compounds with higher antimalarial activity (Tripbenz, Triptosil, Triptiofen, Tripfuroila, and Tripcicloexil) showed no toxicity in HEK293 cells (Supplemental 1).

### 3.4 Effect of Triptiofen in the cell cycle progression of *P. falciparum* 3D7

The impact of Triptiofen (**6**) on the erythrocytic development of *P. falciparum* (3D7) was evaluated after incubation of the compound in the parasite culture at concentrations of 1 and 10 µM for one cycle (48 hours). Parasites development was followed by smears stained with Giemsa every 12 hours. The smears were analyzed under a microscope to assess the distribution of stages (ring, trophozoite, and schizont).

**Figure 4:**
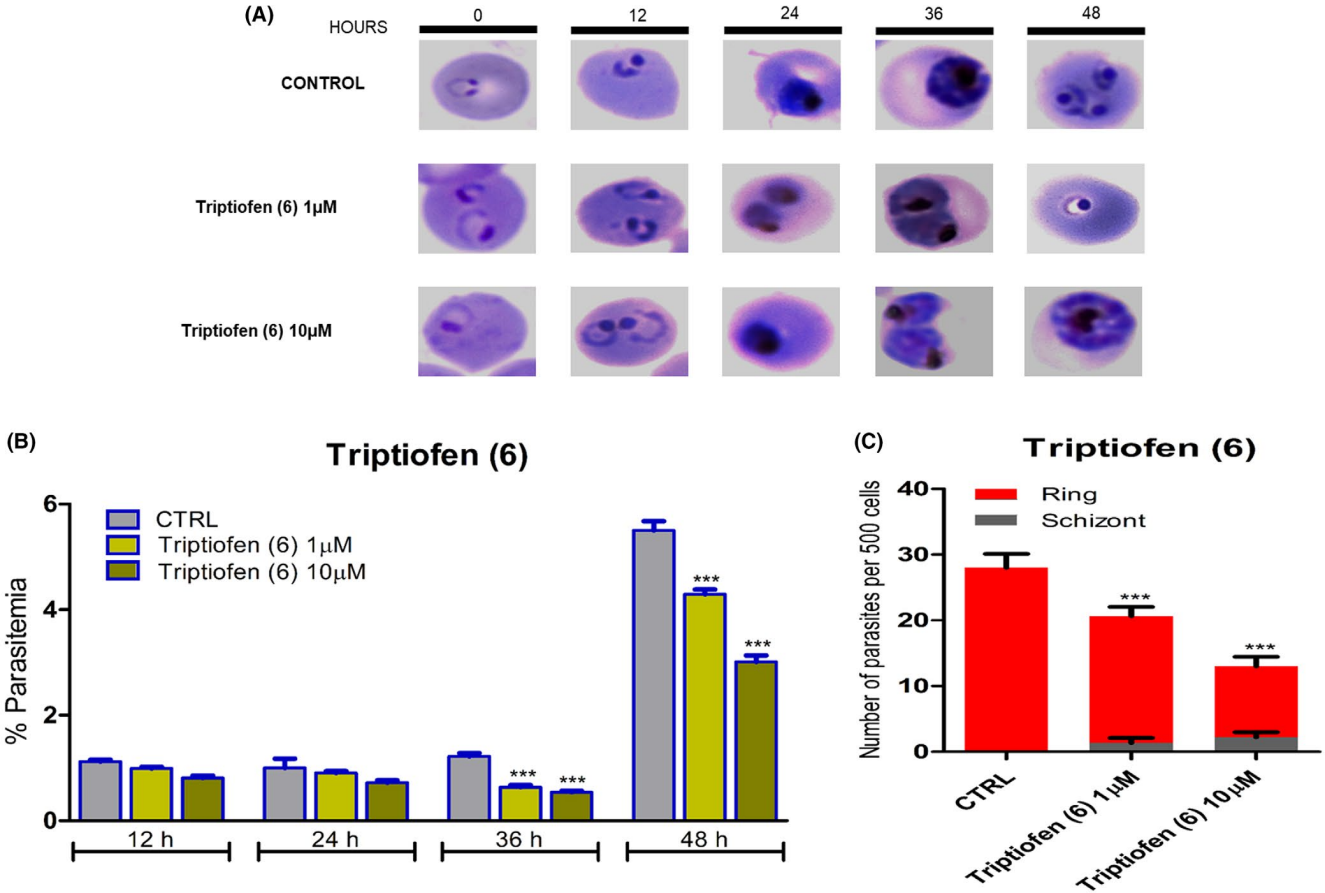
Effect of Triptiofen (**6**) in *Plasmodium falciparum* (3D7) cell cycle progression. Treatment with Triptiofen leads to a dose‐dependent arrest in parasite's intra‐erythrocytic cycle. (A) Smears of Giemsa‐stained of *P. falciparum* (3D7) parasite cultures synchronized and treated with Triptiofen (**6**) (1 and 10 µM). Aliquots taken at the indicated times after compound incubation and images were displayed (B) Parasitemia quantified after 12, 24, 36, and 48 h in the different groups evaluated. (C) Distribution of parasite stages assessed after 48 h displayed as R ring, T trophozoite, and S schizont. “***” indicates a significant difference. One‐way ANOVA followed by Tukey's test (*P ≤ .05; **P ≤ .01; ***P ≤ .001; ****P ≤ .0001)

The results on Figure 4A revealed that after 48 hours, parasites in the control group were able to re‐invade new red blood cells, since the ring stage corresponds to completion of the entire cycle. However, analysis of Giemsa‐smears from parasites treated with Triptiofen (**6**) (1 and 10 µM) showed that although most of the parasites are in the ring stage, we could observe parasites in segmented schizont stage (1.33 ± 0.33 and 2.22 ± 0.32, respectively). Figure 4C shows the distribution of the parasite forms in the solvent control and the groups treated with Triptiofen (1 and 10 µM). Analysis reveals that Triptiofen (**6**) significantly reduces the number of parasites compared to control starting at 36 hours after compound incubation at both concentrations 1 and 10 µM (Figure 4B). The data indicate that Triptiofen acts in mature forms of the parasite.

### 3.5 Effect of indole compounds to block melatonin action in *P. falciparum* parasitemia

**Figure 5:**
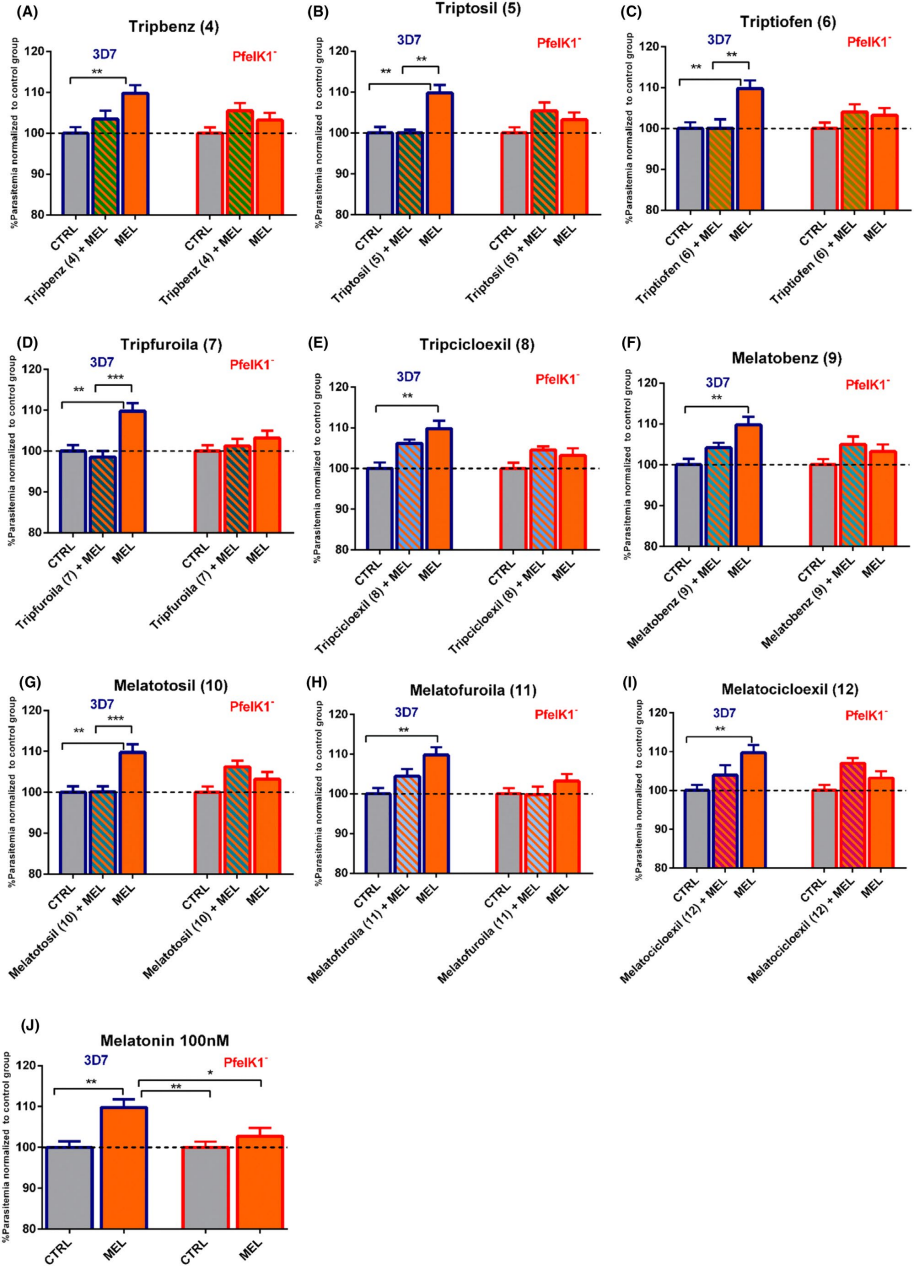
Effect of indole compounds in melatonin action on *Plasmodium falciparum* parasitemia. Erythrocytes infected with *P. falciparum* 3D7 (on the left) or PfeIK1^‐^ (on the right) were incubated for 48 h with 500 nM of each indole compounds Tripbenz (**4**) (A), Triptosil (**5**) (B), Triptiofen (**6**) (C), Tripfuroila (**7**) (D), Tripcicloexil (**8**) (E), Melatobenz (**9**) (F), Melatotosil (**10**) (G), Melatofuroila (**11**) (H), and Melatocicloexil (**12**) (I) along with 100 nM melatonin (MEL). Effect of melatonin in 3D7 and PfeIK1 strains is showed in (J). Parasites were stained with MitoTracker Deep Red and SYBR Green I and final parasitemia was determined by flow cytometer using a BD Accuri C6 (BD Bioscience). Data represent the percentage of parasitemia normalized to the control group treated with solvent (DMSO). Experiments were performed 3 independent times in triplicates. Error bars represent the SEM. “*” indicates significant difference to MEL. One‐way ANOVA followed by Dunnett’s test (*P ≤ .05; **P ≤ .01; ***P ≤ .001; ****P ≤ .0001) or Tukey’s test in (J)

We further evaluate if the compounds were able to abolish the increase in parasitemia triggered by melatonin in *P. falciparum* 3D7 parasites. For this purpose, we performed *in vitro* assays with *P. falciparum‐*infected erythrocytes incubated for 48 hours with 100 nM of melatonin in presence of 500 nM of each synthetic compound. Strikingly, Triptosil (**5**), Triptiofen (**6**), Tripfuroila (**7**), and Melatotosil (**10**) were able to abolish the increase in parasitemia triggered by melatonin in *P. falciparum* 3D7 (Figure 5). Melatonin treatment in presence of Triptiofen (**6**) decreased parasitemia by 10% (100.1% ± 2.15) when compared to treatment with the hormone only (110% ± 1.97) and Tripfuroila (**7**) along with the hormone decreased parasitemia in 11.25% (98.52% ± 1.50).

Although treatment with Melatotosil (**10**) alone resulted in a significant increase in parasitemia (Figure 2G), treatment with this compound along with melatonin abolished the host hormone effect on parasitemia in 3D7 parasites, decreasing parasitemia in 11.69% (98.07% ± 1.21) when compared with treatment with the hormone alone. These data point Melatotosil (**10**) as a potential melatonin receptor agonist, which can act as a partial agonist.

Interestingly, Triptosil (**5**), which differs from Melatotosil (**10**) by the absence of a methoxy group in C5 of the indole ring, was also able to abolish melatonin effect on parasitemia, presenting mean parasitemia of 100% (±0.76) in 3D7, a decrease of 9.7%.

Treatment of PfeIK1^‐^ *P. falciparum* parasites with the indole synthetic compounds along with melatonin has no impact in parasitemia (Figure 5) when compared to control (treated with solvent). Our data confirm that PfeIK1^‐^ knockout *P. falciparum* parasites provide an excellent tool to study the specificity of drugs indol derivatives to block the parasite cycle. The final parasitemia after incubating PfeIK1^‐^ with the compounds was as follows: (Tripbenz (**4**) 105.0 ± (1.82), Triptosil (**5**) 105.4 ± 2.05, Triptiofen (**6**) 104.1 ± 1.87, Tripfuroila (**7**) 102.3 ± 1.53, Tripcicloexil (**8**) 105.5 ± 0.91, Melatobenz (**9**) 105.0 ± 1.97, Melatotosil (**10**) 106.2 ± 1.55, Melatofuroila (**11**) 99.78 ± 2.03, and Melatocicloexil (**12**) 107.0 ± 1.37). These data indicate that PfeIK1 plays an important role in the synchronization pathway triggered by melatonin and its analogs.

### 3.6 Effect of Atovaquone and Artemisinin in PfeIK1^‐^

To further confirm that PfeIK1^‐^ strain is a good model but specific for the indol‐related compounds, we evaluate the susceptibility of PfeIK1^‐^ to classical antimalarials drugs. We performed similar assays with antimalarials by incubating *P. falciparum* 3D7 and PfeIK1^‐^ strains with artemisinin and atovaquone.

**Figure 6:**
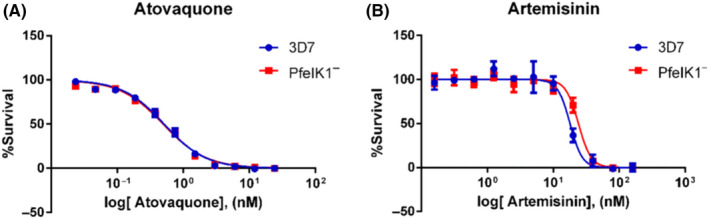
Dose‐response curves of Atovaquone and Artemisinin in *Plasmodium falciparum* 3D7 and PfeIK1^‐^. Erythrocytes infected with *P. falciparum* 3D7 (A and C) or PfeIK1^‐^ (B and D) were incubated with Atovaquone (0.02‐24 nM) or Artemisinin (0.15‐160 nM) for 72 h. Parasitemia was obtained by Flow Cytometry. Experiments were performed three independent times in triplicates. Error bars represent the SEM

Atovaquone is a quinone compound used in therapy against malaria. This compound acts as a competitive inhibitor of ubiquinol, impairing the mitochondrial function by blocking the electron transport chain at the bc1 complex. Inhibition of this complex results in altered concentrations of metabolites in the pyrimidine biosynthesis pathway.^39^ Treatment with atovaquone in PfeIK1^‐^ resulted in IC_50_ 0.47 nM ± 0.07, and this value is very close to the value observed for the wild‐type *P. falciparum* 3D7 (0.40 nM ± 0.08) (Figure 6A). Our data thus confirm our hypothesis of the specificity for the indol, susceptibility of *P. falciparum* lacking the kinase that is still responsive to the drug atovaquone.

Artemisinin and its derivatives are currently employed in first‐line treatment of malaria in combination with other compounds with a different mechanism of action.^2^ This compound contains in its structure an endoperoxide bridge which is cleaved inside the parasite by Fe^2+^ heme, leading to activation of the molecule.^4^ In this activated state, carbon‐centered radicals react with proteins and other biomolecules.^7^ Similarly to the above experiments, parasites PfeIK1^‐^ presented IC_50_ 18.84 nM ± 3.6 for artemisinin, which is compatible with the value found for the wild‐type parasites 3D7, IC_50_ 13.69 nM ± 2.16 (Figure 6B).

### 3.7 Evaluation of calcium response to compounds using *P. falciparum* GCaMP3

We reported that melatonin triggers the synchronization of the *P. falciparum* erythrocytic cycle in a calcium‐dependent manner.^24^ We investigated whether Melatotosil (**10**), which was able to increase parasitemia in *P. falciparum* 3D7, would also trigger a calcium response similar to melatonin.

For this purpose, we performed the calcium assays using a transgenic cell line of *P. falciparum* GCaMP3 parasites that genetically encodes a GFP‐based calcium sensor (GECI),^37^ which increases fluorescence when cytosolic calcium concentration increases. These transgenic parasites are new genetic tools to follow calcium changes within cells as they do not require any dye treatment, which further preserves the cell physiology. Moreover, GECI cells warrant the measurement of cytosolic calcium, thus avoiding the dye compartmentalization.

**Figure 7:**
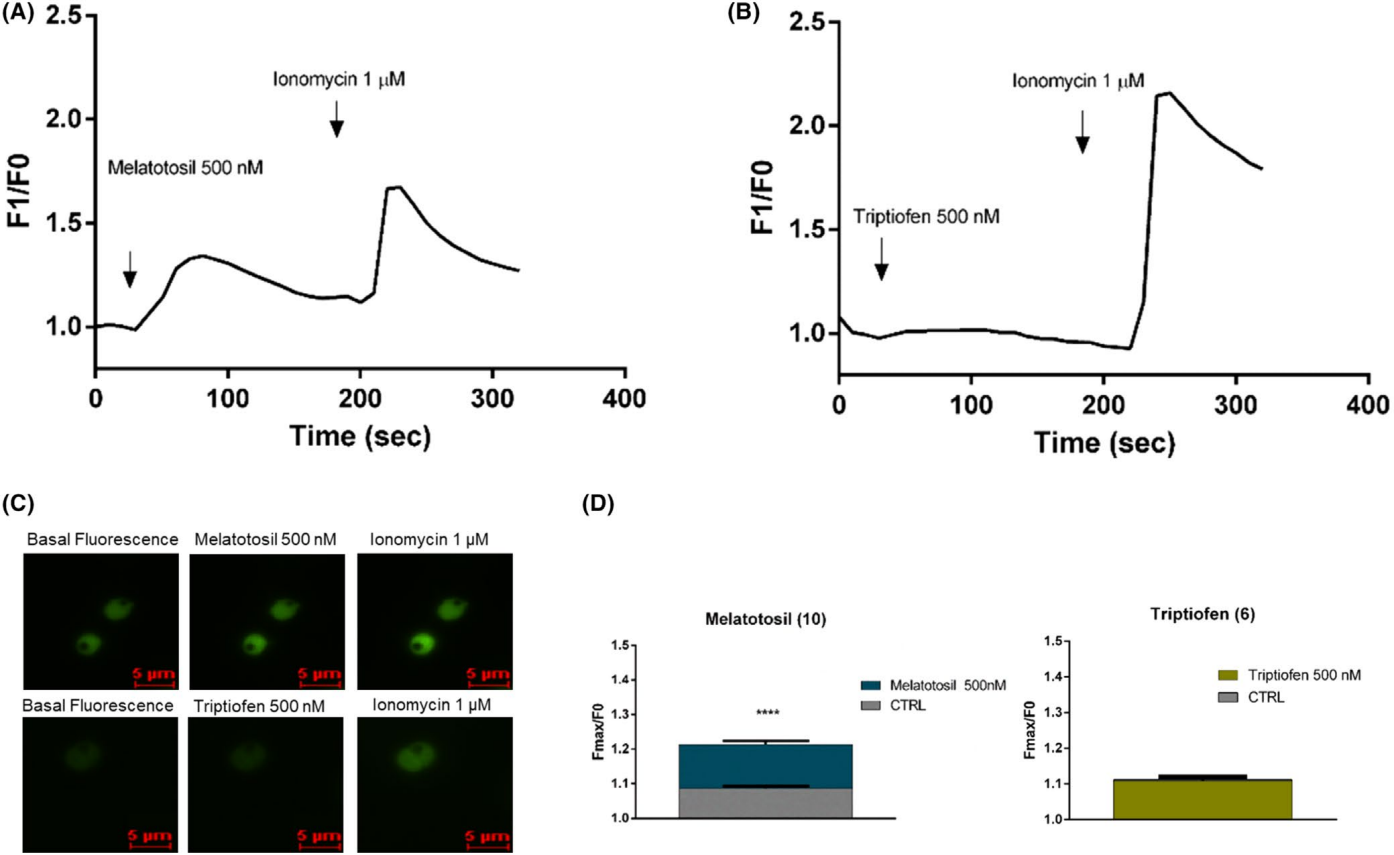
Cytosolic calcium response to Melatotosil (**10**) and Triptiofen (**6**) in PfGCaMP3. Intracellular calcium changes were monitored in a Nikon Eclipse Ti2 microscope. Representative graph of changes in fluorescence with 500 nM of Melatotosil (**10**) (A) and Triptiofen (**6**) (B), normalized to baseline fluorescence is showed. Basal fluorescence (on the left), fluorescence increase after adding Melatotosil (**10**) (in the middle) and fluorescence after adding ionomycin (on the right) is shown in (C). The mean of maximum fluorescence obtained with compound addition normalized to baseline fluorescence is shown (D) for each compound tested. Experiments were performed three independent times in duplicates. Error bars represent the SEM. *Unpaired t test (*P ≤ .05; **P ≤ .01; ***P ≤ .001; ****P ≤ .0001)

By using a microscopy imaging system, we investigated whether the addition of Melatotosil at 500 nM to *P. falciparum* GCaMP3 parasites would be able to trigger a cytosolic calcium response (Figure 7). Our data clearly show that Melatotosil elicits a calcium rise in *P. falciparum* parasites, as control ionomycin at 1 µM concentration was added to ensure the integrity of the cells. We also investigated whether Triptiofen (**6**) was able to induce a calcium response in these parasites. Triptiofen blocked the melatonin effect in 3D7 parasitemia and showed an antimalarial activity. The results obtained showed that Triptiofen 500 nM does not trigger a significant cytosolic calcium rise in these parasites, but ionomycin was still able to induce a calcium rise (Figure 7B). Figure 7D shows the quantification and analysis of 120 cells and the statistical significance of calcium rise for Melatotosil (Fmax/F0 1.21 ± 0.01) but not for Triptiofen (Fmax/F0 1.11 ± 0.008) compared to DMSO (*F*_max_/F0 1.08 ± 0.006; *F*_max_/F0 1.11 ± 0.012 respectively).

## 4 DISCUSSION

Efforts from several laboratories focusing on better understand the rhythms underlying the cell cycle development and coordination to host rhythms of several species of the malaria parasite have been made.

Rijo‐Ferreira et al^40^ evaluated the impact of different factors in parasite rhythms by analyzing the transcriptome of rodent *Plasmodium chabaudi* parasites. They showed that parasites present a group of genes with rhythmic expression sustained in the absence of light, implying that parasites possess an intrinsic clock, and the host is not the only one to drive their cell cycle length. Also, parasites infecting arrhythmic mice experience decay and loss of synchrony, suggesting that a host cue is essential to entrain this clock and synchronize parasites.

Aiming to investigate the rhythmic gene expression in parasites in absence of host cues, Smith et al^41^ analyzed the gene expression from four different strains of *P. falciparum*. The authors showed that *P. falciparum* presents rhythmic gene expression, pointing out an intrinsic oscillator in the parasite.

Another study by Subudhi et al^42^ demonstrated that coordination of the parasite cell cycle to host rhythms plays a central role in the cycling gene expression associated with essential processes for parasite erythrocytic development.

Melatonin and indole derivatives can modulate the *P. falciparum* erythrocytic cycle. The synchronization triggered by melatonin involves different protein kinases such as PfPK7^43^ and PKA^25^ as downstream molecular effectors, although the kinases PfNek‐2 and PfNek‐3 are not involved in synchronization by melatonin.^43^ In this study, we show that an additional protein kinase, PfeIK1, plays a vital role in the synchronization of the erythrocytic cycle in *P. falciparum*. The affirmation is based on the fact that parasites lacking this kinase presented no significant difference in parasitemia after melatonin treatment.

Little is known of the PfeIK1 role in the *Plasmodium* life cycle. Fennel et al^28^ has previously reported that PfeIK1 phosphorylates the eukaryotic initiation factor 2 in its α subunit (eIF 2α) in response to aminoacid starvation. Lima et al^44^ investigated the effect of melatonin in the gene expression of 3D7 and PfPK7^‐^ by RNA‐seq. The authors observed 38 genes modulated by melatonin in 3D7 parasites, including the gene transcribing the eIF2, which is phosphorylated by Protein Kinases of the initiation factor 2 of *P. falciparum*, such as PfeIK1. The data point out the importance of regulation of protein translation for the melatonin effect. Moreover, our data contribute to decodes the potential role of PfeIK1 in melatonin‐signaling pathways on parasite synchronization.

In this work, we aimed to (a) investigate whether PfeIK1 plays a role in the melatonin pathway on parasite synchronization promoting cell cycle modulation and (b) searching for new forms to block the parasite cell cycle by using new indol‐related compounds. To achieve the goal, we used PfeIK1 knockout strain parasites and a novel series of synthetic compounds.

The relevance of these studies relies on the fundamental question on how the parasite transduces the external signaling and puts in action its molecular machinery involved in the melatonin pathway leading to rhythmic development and synchronization of parasites erythrocytic cycle. Moreover, there is an urgency to find new ways to treat malaria since parasite resistance to the classical antimalarials, such as chloroquine and artemisinin, is well known in several continents.^3^, ^4^, ^5^, ^6^, ^7^, ^8^, ^9^, ^10^ Thus, studies searching for new ways of treating malaria are required. Svogie et al showed the indolyl‐3‐ethanone‐α‐thioesters as a potential class of antimalarial, with IC_50_ values in the micromolar range. Luthra et al identified an indole compound (2j) able to disrupt the melatonin‐induced synchronization *in vitro* in *P. falciparum*. In the same line, Lunga et al tested a library of indole compounds as potential antimalarials.^36^, ^45^, ^46^ Other indole compound, indolmycin, inhibit the protein translation in *P. falciparum* apicoplast by inhibiting the tryptophanyl‐tRNA synthetase leading to a delayed death of the parasite.^47^, ^48^

Since PfeIK1^‐^ parasites were susceptible to indole derivatives, we also investigated if PfeIK1 knockout would interfere with susceptibility of parasite against classical antimalarials. Zhang et al^49^ have reported that parasite treatment with artemisinin resulted in an increase in eIF2 phosphorylation by PK4, and inhibition of this kinase can avoid that parasites enter in latency. The results obtained here show that PfeIK1^‐^ reacts similarly to wild‐type parasites 3D7 under mitochondrial and oxidative stress caused by atovaquone and artemisinin. These data indicate that the lack of PfeIK1 does not interfere with parasites’ susceptibility to atovaquone and artemisinin.

Next, we evaluated the new indole compounds' ability to interfere in the melatonin pathway and impair synchronization in *P. falciparum*. We identified Melatotosil (**10**) as a potential melatonin receptor partial agonist, since the compound increased parasitemia in 3D7 and was also able to inhibit melatonin action in 3D7. Interestingly, Triptosil (**5**), which differs from Melatotosil (**10**) by the absence of a methoxy group in C5 of the indole ring, showed no effect in 3D7 parasitemia but could abolish melatonin effect.

Until now, a canonical melatonin receptor has not been identified in *Plasmodium* database, although by bioinformatics analysis, four candidates for serpentine‐like receptors were identified.^50^ The finding of the molecular identity of such receptor in *Plasmodium* is of great relevance, given the role of melatonin on parasite kinases. Vertebrate Melatonin receptors are G‐protein‐coupled receptors present in all tissues and were initially divided into three subfamilies MT1, MT2, and the orphan GPR50. MT1 and MT2 activation result in inhibition of cAMP production; also, activation of MT1 can result in inositol‐phosphate production.^51^ Structure‐activity studies about melatonin receptors showed that the 3‐acylaminoethyl and 5‐methoxy groups play an essential role in receptor binding and activation. However, the 5‐methoxy group's presence is not critical to receptor binding, since the competitive agonist Luzindole lacks the methoxy group.^18^, ^52^

As expected, the treatment of PfeIK1^‐^ parasites with the indole synthetic compounds and melatonin has no impact on parasitemia compared to control (treated with solvent). Our data confirm that PfeIK1^‐^ knockout *P. falciparum* parasites provide an excellent tool to study the specificity of indol derivatives to block the parasite cycle.

Our data indicate that PfeIK1 plays a role in parasite synchronization, showing that melatonin pathways involved at synchronization and cell cycle progression are two distinct pathways. The kinase PfeIK1 is central in the parasite synchronicity by melatonin.

## CONFLICT OF INTEREST

The authors declare no conflict of interest.

## AUTHOR CONTRIBUTIONS

BKMD, MN, MRRA, DPP , BMS, FCSA, RY and DS performed and designed the experiments AKJ and CRSG are the principal investigators who contributed for the design of the experiments and data discussion as well as managed the project. All the authors contributed to the writing of the paper and interpretation of the data.

## Supporting information

Supplementary Material
