# Supplementary figures and images for: The Plasmodium falciparum eIK1 kinase (PfeIK1) is central for melatonin synchronization in the human malaria parasite. Melatotosil blocks melatonin action on parasite cell cycle
Source: J Pineal Res. 2020 Aug 7;69(3):e12685. doi: 10.1111/jpi.12685 (PMC7539967; doi:10.1111/jpi.12685)

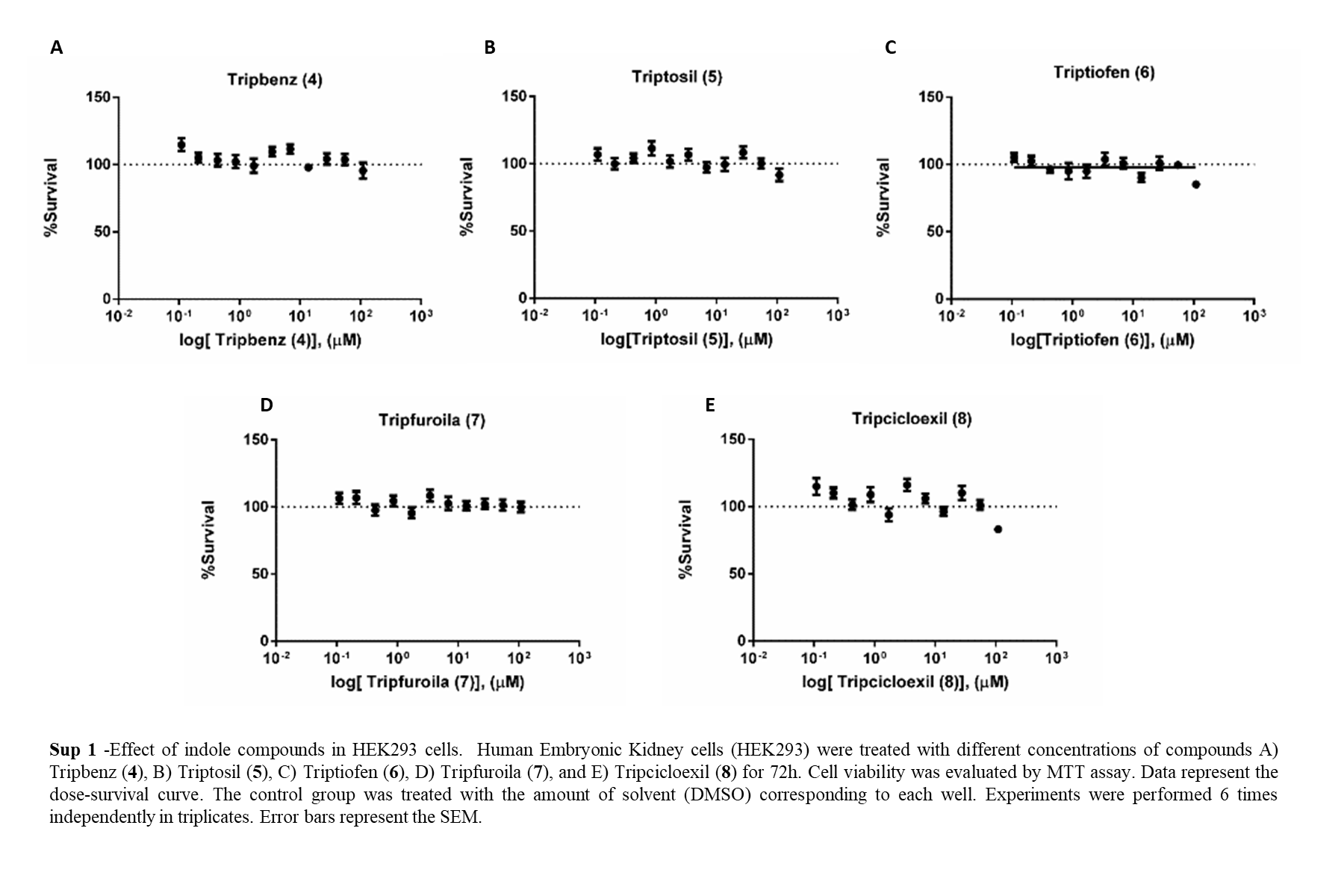

Supplement: Supplementary file 1 — Supplementary Material [file JPI-69-e12685-s001.tif]
